# Cybervictimization, Depression, and Adolescent Internet Addiction: The Moderating Effect of Prosocial Peer Affiliation

**DOI:** 10.3389/fpsyg.2020.572486

**Published:** 2020-09-29

**Authors:** Zhenhai Wang, Qi Xie, Mucheng Xin, Chang Wei, Chengfu Yu, Shuangju Zhen, Sha Liu, Jianping Wang, Wei Zhang

**Affiliations:** ^1^Center for Studies of Psychological Application, School of Psychology, South China Normal University, Guangzhou, China; ^2^Department of Psychology and Research Center of Adolescent Psychology and Behavior, School of Education, Guangzhou University, Guangzhou, China; ^3^School of Health Management, Guangzhou Medical University, Guangzhou, China; ^4^School of Politics and Public Administration, South China Normal University, Guangzhou, China

**Keywords:** cybervictimization, internet addiction, depression, prosocial peer affiliation, adolescent

## Abstract

Although vast research has shown that cybervictimization is a significant risk factor of adolescent’s internet addiction (IA), little is known about the mediating and moderating mechanisms behind this relationship. The current study examined whether depression mediated the relationship between cybervictimization and adolescent’s IA, and whether the direct and indirect effect was moderated by prosocial peer affiliation (PPA). A sample of 1,006 adolescents (*Mean_age_* = 13.16; *SD* = 0.67) anonymously completed the questionnaires. The results revealed that the positive association between cybervictimization and adolescent’s IA was mediated by depression. Moderated mediation analysis further showed that PPA moderated the association between cybervictimization and adolescent’s IA. However, this indirect effect was stronger for adolescents with high PPA than for those with low PPA, which means that the protective effects of PPA are limited. These findings highlight the mediating and moderating mechanisms between cybervictimization and adolescent’s IA, and provide guidance for the prevention and intervention in adolescent’s IA.

## Introduction

According to the 44th statistical report on internet development in China [China Internet Network Information Center (CNNIC), 2019], the number of Chinese internet users has reached 854 million, of which 16.9% are aged between 10 and 19. While the internet offers significant convenience, the impact of the internet on social and mental health has increasingly become a concern, especially the negative impact of internet addiction (IA; Young and De Abreu, 2011). IA can generally be conceptualized as the incapacity to control own use of the internet, which may have negative repercussions in daily life, such as withdrawal symptoms, low tolerance of stress, and scholastic or occupational impairment (Block, 2008; Young and De Abreu, 2011). Compared to adults, adolescents are more susceptible to IA because they have higher sensation seeking and poor self-control (Spada, 2014). Many empirical studies indicate that IA is significantly associated with adolescents’ emotional and behavioral problems, such as substance abuse, sleep problems, and suicidal behaviors (Gamez-Guadix et al., 2015; Cheng et al., 2018; Alimoradi et al., 2019). To develop practical prevention and remedial programs, it is necessary to identify the risk and protective factors and the underlying mechanisms of IA in adolescents.

### The Relationship Between Cybervictimization and Adolescent Internet Addiction

Cybervictimization, as a new form of victimization, has received increased attention over the past few years in terms of its effect on adolescents’ IA. In this context, cyberbullying is defined as the behavior of individuals or groups repeatedly sending hostile or offensive messages through electronic or digital media, with the intention of causing harm or discomfort to others, while cybervictimization refers to being a victim of this behavior (Tokunaga, 2010). As the internet technology has become more widespread, the prevalence of cybervictimization has been increasing among adolescent (Kowalski et al., 2018). A recent study on the prevalence of cybervictimization shows that 65.0% of adolescents have suffered cybervictimization at least once in their lifetime (Brochado et al., 2017). Compared with traditional victimization, cybervictimization involves more extreme violations of personal privacy, coupled with the perpetrators ability to remain anonymous as well as to harass others without being constrained by time and place (Kowalski et al., 2018), which may lead to more psychological and behavioral problems for the victim (Sourander et al., 2010). Some empirical evidence has supported the view that cybervictimization has a high correlation with adolescents’ IA (Gamez-Guadix et al., 2013; Chang et al., 2015; Lin et al., 2020). For example, using a longitudinal study, Gamez-Guadix et al. (2013) found that cybervictimization significantly predicted problematic internet use 6 months later among adolescents. Similarly, Chang et al. (2015) found that cybervictimization was highly related to adolescents’ IA. These findings highlight that being a victim of cybervictimization can put adolescents at risk of IA.

Although vast research has shown a positive association between cybervictimization and adolescents’ IA, the mediating and moderating processes involved in this link are still largely unclear. It is necessary to explore these factors to provide more effective interventions to reduce adolescents’ IA.

### The Mediating Role of Depression

Previous studies have shown that adolescents who suffer from cybervictimization often exhibited a series of psychological problems, such as anxiety, loneliness, low self-esteem, and depression (Guo, 2016). However, among the many adverse consequences of cybervictimization, depression seems to be one of the most common and important (Kowalski et al., 2014; Kwan et al., 2020). Both previous cross-sectional and longitudinal studies can provide evidences that cybervictimization is an important risk factor for depressive symptoms (Landoll et al., 2015; Calvete et al., 2016; Chu et al., 2018), which means that adolescents are more likely to develop depressive symptoms after suffering from cybervictimization. According to the self-medication hypothesis of addictive disorders (Khantzian, 1985), addictive behaviors are considered as a maladaptive response when an individual copes with negative emotions or stressful states. Given this theory, adolescents with more depressive symptoms are more likely to eliminate negative emotions through IA. Therefore, it is reasonable to assume that psychological disorders such as depression may mediate the relationship between cybervictimization and adolescents’ IA. Consistent with this theoretical framework, several empirical research have demonstrated this view (Zhao et al., 2017; Gao et al., 2018; Sela et al., 2020). For example, a study of 10,000 Chinese vocational school students showed that depression significantly mediated the relationship between negative life events and IA (Zhao et al., 2017). However, as far as we know, the mediating role of depression between cybervictimization and IA has not been directly tested.

First, adolescents who suffer from cybervictimization are more likely to develop depressive symptoms. Specifically, since cybervictimization always involves verbal insults and attacks on one’s personal values, it often reduces adolescents’ self-esteem and increases their sense of inadequacy (Gamez-Guadix et al., 2013; Cole et al., 2016), which in turn leads to depression. In addition, adolescents who suffer intentional and repeated harassment may have more social anxiety and difficulty adapting (Cenat et al., 2018; Wang et al., 2019), which increases their risk of depression. Some previous studies provide evidence for this view (Gamez-Guadix et al., 2013; Li et al., 2018; Wright, 2018). For example, longitudinal studies have found that, over time, young people develop depressive symptoms due to cybervictimization (Gamez-Guadix et al., 2013; Wright, 2018). Similarly, a study of 793 students aged 11–19 years found by Li et al. (2018) that cybervictimization was a significant predictor of depressive symptoms.

Second, adolescents with higher levels of depression are more likely to develop IA. This may be because adolescents are likely to satisfy their psychological needs and escape reality through problematic internet use when they experience depression (Zhao et al., 2017; Sela et al., 2020). Likewise, Anand et al. (2018) concluded that the usage of the internet may be a strategy, by which adolescents can cope with negative emotions caused by cybervictimization, which in turn increases their dependence on the internet. In view of the above literature, it is reasonable to expect that suffering from cybervictimization may be related to depression, which in turn is associated with adolescents’ IA.

### The Moderating Role of Prosocial Peer Affiliation

Although suffering from cybervictimization may enhance the risk of IA through depression, it seems not necessarily that all adolescents will develop depression and IA when they are exposed to cybervictimization (Fisher et al., 2016; Baldry et al., 2019). According to the risk and resilience framework (Masten, 2001), this difference may be due to protective factors. Adolescents spend less time with their families and more time with their peers (Tian et al., 2019; Bao et al., 2020). Given peers’ growing influence, it is necessary to explore the influence of peer factors [e.g., prosocial peer affiliation (PPA)] on adolescent development. PPA often means establishing and maintaining relationships with peers who perform voluntary behaviors intended to benefit others, such as volunteering, donating, mentoring troubled peers, and valuing good grades (Fabes et al., 2012).

First, as a positive and supportive peer relationship, PPA could provide emotional and behavioral support for adolescents, which can help compensate for the negative impact of adverse experiences such as cybervictimization (Han and Margolin, 2016; Healy and Sanders, 2018). For example, Rusby et al. (2019) have found that the negative impact of relational victimization may be lessened by spending time with prosocial peers. Moreover, a systematic review showed that positive peer interaction was a very strong protective factor against being a victim of cyberbullying (Zych et al., 2019).

Second, according to the social learning theory (Bandura, 1977), the behaviors of individuals are influenced by their peers, which means that adolescents who observe prosocial behaviors of peers are likely to have more similar behaviors and fewer problem behaviors. Therefore, it is reasonable to expect that affiliation with prosocial peers can reduce adolescents’ deviant behaviors by encouraging participation in prosocial behaviors and preventing relationships with deviant peers. Consistent with this theory, many empirical studies indicate that adolescents with a high proportion of prosocial peers are less likely to engage in substance use and delinquency (Barry and Wentzel, 2006; Han and Margolin, 2016). More importantly, research has also shown that PPA can significantly buffer personal and environmental risk factors on adolescents’ academic, emotional, and behavioral adjustment (Burt and Klump, 2014; Han and Margolin, 2016; Mason et al., 2019). For example, an empirical study of 500 pairs of twins showed that the genetic influence on rule-breaking behavior was several times greater in those with low-level PPA than in those with high-level PPA (Burt and Klump, 2014). Moreover, other research has shown that prosocial peer network moderated the effects of depression on substance use (Mason et al., 2019). Therefore, it is reasonable to expect that PPA can moderate the direct and indirect link between cybervictimization and adolescents’ IA.

### The Present Study

Based on the self-medication hypothesis of addiction (Khantzian, 1985) and the risk and resilience framework (Masten, 2001), we propose the following.

**Figure 1:**
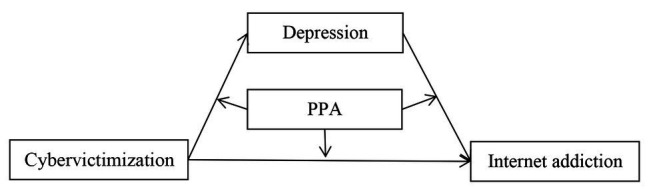
The proposed mediated moderation model. PPA, prosocial peer affiliation.

*Hypothesis* 1: Depression can mediate the link between cybervictimization and adolescent IA.
*Hypothesis* 2: PPA moderates the direct and indirect links between cybervictimization and adolescents’ IA. Figure 1 illustrates the proposed research model.

## Materials and Methods

### Participants

Participants were recruited from three junior middle schools in Guangdong province in south China, using the method of random cluster sampling (Teddlie and Yu, 2007). The sample was stratified by city size (large, medium, and small cities). A total of 1,006 adolescents (51.79% female), whose ages ranged from 12 to 16 (*Mean_age_* = 13.16, *SD* = 0.67) participated. There were 556 seventh graders and 450 eighth graders. Reflecting the demographics of the sample, 48.63% of participants’ fathers and 54.07% of their mothers have less than a high school education.

### Measures

#### Cybervictimization

Cybervictimization was assessed with the cyberbullying victimization scale (Erdur-Baker and Kavsut, 2007). Participants indicated the frequency they had experienced each of the 18 cybervictimization behaviors during the past 6 months (e.g., “Someone spread rumors about me online”) on a four-point scale ranging from 1 (never) to 4 (more than five times). Responses across the 18 items were averaged, with higher scores reflecting more experience of cybervictimization. The result of confirmatory factor analysis (CFA) indicates the scale has good structure validity in this study: *χ*^2^/*df* = 5.01, CFI = 0.88, RMSEA = 0.063. Moreover, in the current study, the measure demonstrated very good reliability (*α* = 0.82).

#### Internet Addiction

Internet addiction was measured by a nine-item scale adapted from the internet gaming disorder questionnaire (Pontes and Griffiths, 2015). Participants indicated how often they feel dependent on the internet (e.g., “Do you systematically fail when trying to control or cease your internet use?”) on a three-point scale ranging from 1 (never) to 3 (often). Responses across the nine items were averaged, with higher scores reflecting a higher tendency to IA. The result of CFA indicates the scale has good structure validity in this study: *χ*^2^/*df* = 2.32, CFI = 0.97, RMSEA = 0.036. Moreover, in the current study, the measure demonstrated good reliability (*α* = 0.74).

#### Depression

Depression was measured by the Center for Epidemiologic Studies Depression Scale (CES-D; Radloff, 1977). Participants indicated how often they experienced depressive symptoms over the past week (e.g., “I felt that everything I did was an effort”) on a four-point scale ranging from 1 (never) to 4 (always). Responses were averaged across all items, with higher scores indicating more depressive symptoms. The result of CFA indicates the scale has good structure validity in this study: *χ*^2^/*df* = 5.25, CFI = 0.89, RMSEA = 0.065. Moreover, in the current study, the measure demonstrated excellent reliability (*α* = 0.88).

#### Prosocial Peer Affiliation

Prosocial peer affiliation was assessed with five items adapted from prior published questionnaires (Metzler et al., 1998; Walden et al., 2004). Peers’ prosocial behaviors include helping others, cooperating, sharing with others, working hard in school, and volunteering. Participants indicated how many of their friends had shown each of the five prosocial behaviors during the past 6 months (e.g., “How many of your friends helped others in the past 6 months”) on a six-point scale ranging from 1 (none) to 6 (almost all). Responses were averaged across all items, with higher scores reflecting greater PPA. The result of CFA indicates the scale has good structure validity in this study: *χ*^2^/*df* = 7.09, CFI = 0.99, RMSEA = 0.078. Moreover, for this study, the measure demonstrated outstanding reliability (*α* = 0.94).

### Control Variables

Given that adolescents’ gender, age, and impulsivity are significant influencing factors in IA (Spada, 2014; Zhu et al., 2016; Cheng et al., 2018), we controlled for these variables in the statistical analyses. Impulsivity was measured using the UPPS-P Scale (Cyders et al., 2014). Participants indicated their responses on a four-point scale ranging from 1 (strongly disagree) to 4 (strongly agree). Responses were averaged across all items, with higher scores reflecting higher impulsivity. The result of CFA indicates the scale has good structure validity in this study: *χ*^2^/*df* = 4.85, CFI = 0.90, RMSEA = 0.062. Moreover, for this study, the measure demonstrated excellent reliability (*α* = 0.82).

### Procedure

This research was approved by the Ethics Review Committee of the School of Education, Guangzhou University. Before collecting the information, we obtained the informed consent of teachers, parents, and the participating adolescents. Participating adolescents took about 30 min to complete a series of self-report questionnaires in their regular classrooms. All measures were implemented by experienced psychology graduate students using standardized guidance that was prepared by the researcher. To encourage honest responding, participants were informed that their responses would be strictly confidential and that their participation was voluntary.

### Statistical Analyses

In primary analyses, SPSS 25.0 version was used to conduct descriptive statistics and correlations for all variables. We used Mplus 8.1 to conduct a structural equation modeling to test whether the impact of cybervictimization on adolescent’s IA was mediated by depression and whether PPA can moderated this indirect link, using maximum likelihood estimation and bias-corrected percentile bootstrapping with 1,000 replications. In these analyses, we controlled for gender, age, and impulsivity by entering them as predictor variables into regression equations. The missing data were less than 2% and were handled by mean substitution.

## Results

### Preliminary Analyses

The means, standard deviations, and correlation coefficients for all variables in this study are shown in Table 1. The results showed that cybervictimization is positively correlated with depression and IA. Additionally, depression is negatively correlated with IA. Moreover, PPA is negatively correlated with depression and IA.

**Table 1:** Descriptive statistics and correlations for all variables.

Variables	1	2	3	4	5	6
1. Age						
2. Impulsivity	−0.08^*^					
3. Cybervictimization	0.00	0.21^***^				
4. PPA	−0.02	−0.26^***^	−0.09^**^			
5. Depression	0.05	0.47^***^	0.29^***^	−0.29^***^		
6. IA	0.02	0.35^***^	0.30^***^	−0.16^***^	0.37^***^	
*Mean*	13.16	2.12	1.13	4.06	1.71	1.26
*SD*	0.67	0.40	0.20	1.03	0.48	0.28

PPA, prosocial peer affiliation; IA, internet addiction. ^*^p < 0.05, ^**^p < 0.01, ^***^p < 0.001.

### Testing for the Mediating Effect of Depression

**Figure 2:**
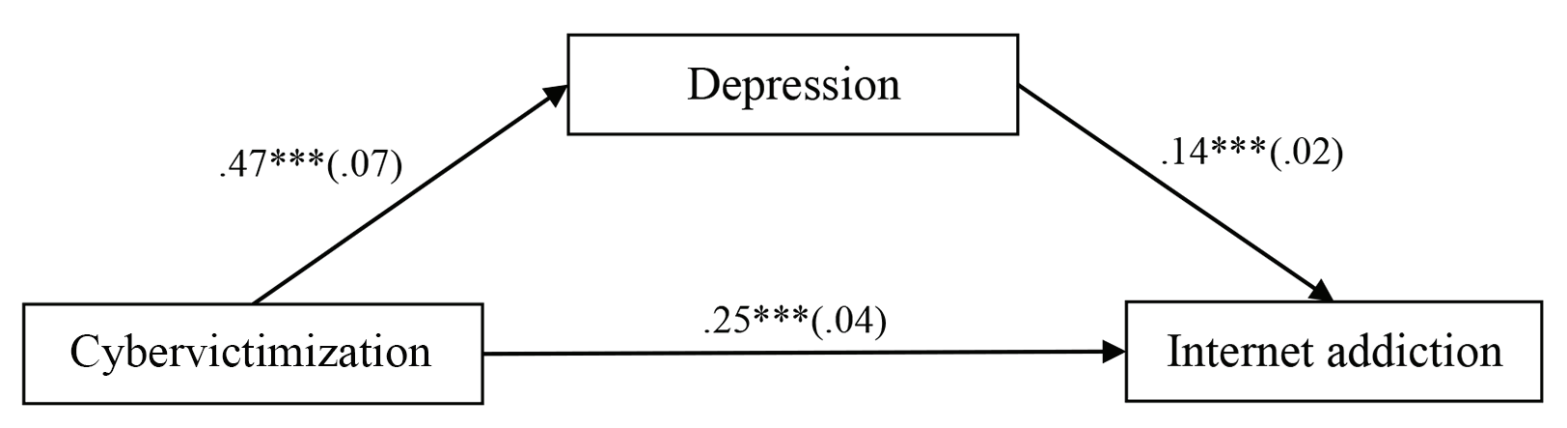
Model of the mediating role of depression between cybervictimization and internet addiction (IA). Values are unstandardized coefficients (outside the brackets) and the standard error is enclosed in parentheses. Paths between gender, age, impulsivity, and each of the variables in the model are not displayed. Of those paths, the following were significant: gender [*b* = −0.15, *SE* = 0.03, 95% CI (−0.20, −0.10)], age [*b* = 0.07, *SE* = 0.02, 95% CI (0.03, 0.10)], and impulsivity [*b* = 0.53, *SE* = 0.03, 95% CI (0.46, 0.59)] to depression; gender [*b* = 0.06, *SE* = 0.02, 95% CI (0.03, 0.09)] and impulsivity [*b* = 0.14, *SE* = 0.02, 95% CI (0.10, 0.18)] to IA. ^***^p < 0.001.

First, this study tested the direct effect (total effect “c”) between cybervictimization and IA. After controlling for age, gender, and impulsivity, it was shown that cybervictimization had a significant direct effect on IA [*b* = 0.32, *SE* = 0.04, 95% CI (0.23, 0.41)], with the explained variance *R*^2^ = 0.18. Next, we further tested the mediation model. The mediation model represented in Figure 2 revealed an excellent fit to the data: *χ*^2^/*df* = 1.81, CFI = 1.00, RMSEA = 0.016. As in Figure 2, cybervictimization positively predicted depression [*b* = 0.47, *SE* = 0.07, 95% CI (0.35, 0.60)], and depression positively predicted IA [*b* = 0.14, *SE* = 0.02, 95% CI (0.10, 0.17)]. Moreover, the residual effect of cybervictimization on IA was significant [*b* = 0.25, *SE* = 0.04, 95% CI (0.17, 0.33)], and the explained variance (*R*^2^) are 0.29 for depression and 0.22 for IA. Furthermore, bias-corrected percentile bootstrapping analyses indicated that depression significantly mediated the link between cybervictimization and adolescent IA [indirect effect = 0.06, *SE* = 0.01, 95% CI (0.04, 0.10)].

### Testing for Moderated Mediation

**Figure 3:**
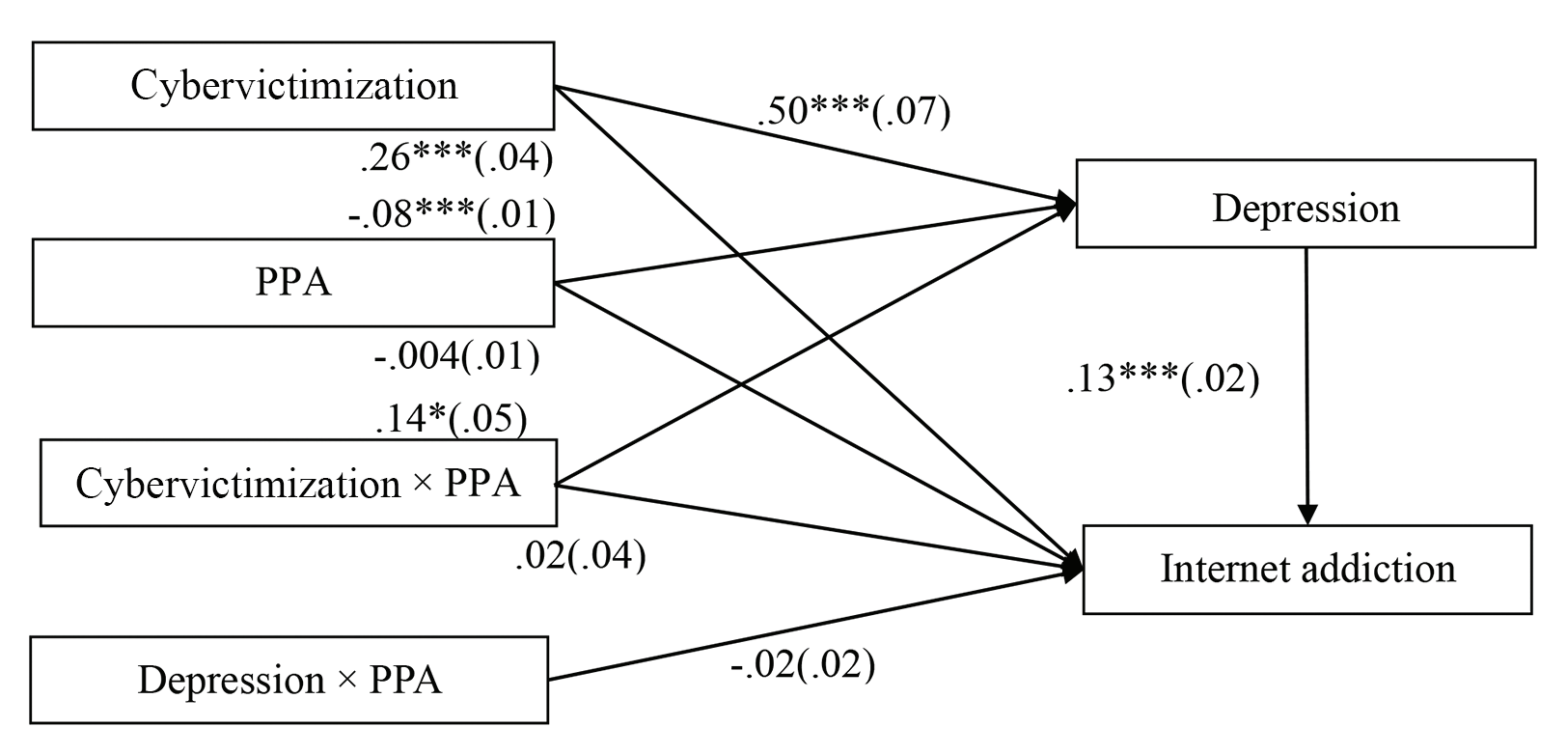
Model of the moderating role of PPA on the indirect relationship between cybervictimization and IA. PPA, prosocial peer affiliation. Values are unstandardized coefficients (outside the brackets) and the standard error is enclosed in parentheses. Paths between gender, age, impulsivity, and each of the variables in the model are not displayed. Of those paths, the following were significant: gender [*b* = −0.16, *SE* = 0.03, 95% CI (−0.21, −0.11)], age [*b* = 0.06, *SE* = 0.02, 95% CI (0.02, 0.10)], and impulsivity [*b* = 0.47, *SE* = 0.03, 95% CI (0.41, 0.54)] to depression; gender [*b* = 0.06, *SE* = 0.02, 95% CI (0.03, 0.09)] and impulsivity [*b* = 0.14, *SE* = 0.02, 95% CI (0.10, 0.19)] to IA. ^*^p < 0.05, ^***^p < 0.001.

**Figure 4:**
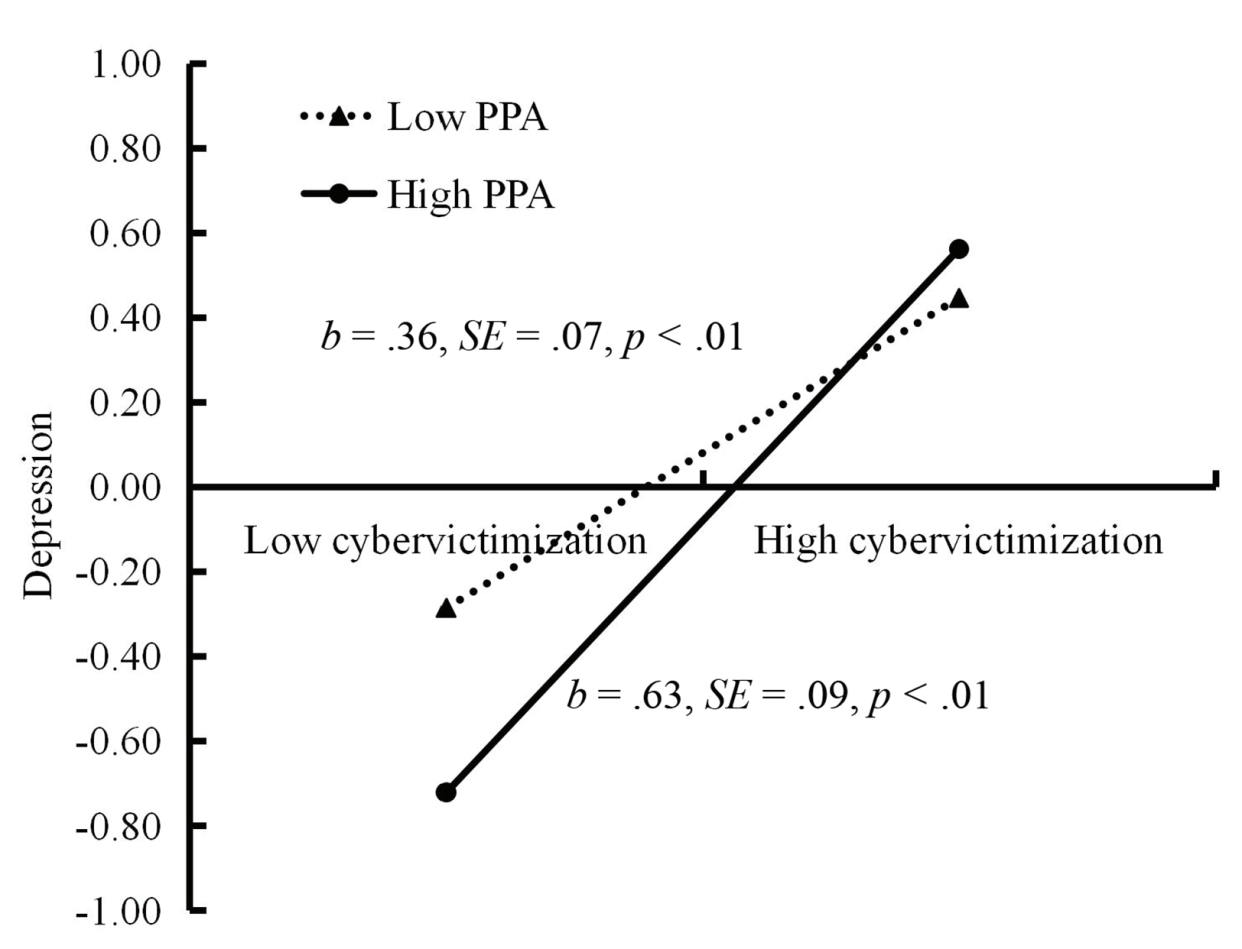
Depression among adolescents as a function of cybervictimization and PPA.

The moderated mediation model represented in Figure 3 revealed a good fit to the data: *χ*^2^/*df* = 3.40, CFI = 0.96, RMSEA = 0.067. The bias-corrected percentile bootstrapping results indicated that the indirect effect of cybervictimization on adolescent IA through depression was moderated by PPA. Specifically, PPA moderated the association between cybervictimization and depression [*b* = 0.14, *SE* = 0.05, 95% CI (0.03, 0.24)]. We conducted a simple slopes test, and, as depicted in Figure 4, adolescents who reported higher PPA (1 *SD* above *M*) experienced less depression compared to those who reported lower PPA (1 *SD* below *M*) when experiencing low levels of cybervictimization; however, the difference between these two groups was non-significant when cybervictimization was high [1 *SD* above *M*; *b* = 0.63, *SE* = 0.09, 95% CI (0.45, 0.82)] than for those who reported lower PPA [1 *SD* below *M*; *b* = 0.36, *SE* = 0.07, 95% CI (0.22, 0.51)]. Moreover, PPA had a significant negative association with depression [*b* = −0.08, *SE* = 0.01, 95% CI (−0.10, −0.06)]. However, the interaction between cybervictimization and PPA in predicting IA [*b* = 0.02, *SE* = 0.04, 95% CI (−0.05, 0.09)] was not significant. Moreover, the interaction between depression and PPA in predicting IA [*b* = −0.02, *SE* = 0.02, 95% CI (−0.05, 0.02)] was also not significant. The explained variance (*R*^2^) is 0.32 for depression and 0.22 for IA.

The bias-corrected percentile bootstrapping method was used to examine the conditional indirect effects of cybervictimization on IA as a function of PPA. Specifically, the indirect association between cybervictimization and IA *via* depression was stronger for adolescents with high PPA [indirect effect = 0.08, *SE* = 0.02, 95% CI (0.04, 0.13)] than for those with low PPA [indirect effect = 0.05, *SE* = 0.02, 95% CI (0.02, 0.10)]. Therefore, the mediating effect of depression between cybervictimization and adolescent IA was moderated by PPA.

## Discussion

Although there is growing evidence of the adverse effects of cybervictimization on adolescent adaptation (Aricak and Ozbay, 2016; Palermiti et al., 2017; Kwan et al., 2020), little research has examined its mediating and moderating mechanisms. To address this gap, this study investigated whether depression mediates the relationship between cybervictimization and adolescent’s IA, and whether this mediating effect is moderated by PPA. Investigating these mechanisms can help identify effective interventions for reducing the risk of adolescent’s IA.

### The Mediating Role of Depression

In general, our study found that cybervictimization is significantly positively corelated to IA, which means that suffering from cybervictimization is an important risk factor for adolescent’s IA. Furthermore, consistent with hypothesis 1, our results found that depression can partly mediate the relationship between cybervictimization and adolescents’ IA, which reveals that depression is an important potential psychosocial factor that helps explain why strong cybervictimization is associated with more IA. This finding supports the self-medication hypothesis of addictive disorders (Khantzian, 1985) that posits that addictive behaviors may be a compensatory behavior to deal with psychological problems such as depression and reduce psychological distress. Although extensive studies have investigated the association between cybervictimization and depression, as well as the association between depression and IA (Lee et al., 2014; Li et al., 2018; Wang et al., 2019), the present study is the first to highlight depression as a critical mediator of the negative impact of cybervictimization on adolescent’ IA.

Beside the overall mediation results, each of the specific relationships in our mediation model is valuable. First, our research found that cybervictimization is significantly positively correlated with depression, which supports previous research (Gamez-Guadix et al., 2013; Li et al., 2018) and the view that it is particularly important to protect adolescents from cybervictimization (Kowalski et al., 2018; Zych et al., 2019). More specifically, as an important risk factor highly related to adolescent depression, cybervictimization usually involves insults to body image and personal values, and further leads to negative emotions, psychological distress, and depression (Calvete et al., 2016).

Moreover, the second part of the mediation chain also highlights the previously supported relations between depression and adolescent’s IA (Zhao et al., 2017; Gao et al., 2018). Specifically, depressed adolescents tend to have more negative self-evaluations and experience stronger feelings of worthlessness (Yen et al., 2019). Consequently, depressed adolescents are more likely to relieve their negative emotions or escape from their frustrations by surfing the internet, playing internet games, watching internet videos, and so on, which may aggravate their addiction to the internet (Anand et al., 2018; Yucens and Uzer, 2018). Therefore, being aware of the role of depression in the relationship between cybervictimization and adolescent’ IA is very important for prevention and intervention.

Taken together, our research proves that depression is not only a possible adverse consequence of cybervictimization but also closely related to adolescents’ IA. Notably, depression only partially mediates the relationship between cybervictimization and IA. Thus, there may be other factors (such as psychological security) that should be considered in the mediating process.

### The Moderating Effect of Prosocial Peer Affiliation

One valuable findings of the present study is that the indirect relationship between cybervictimization and IA through depression is moderated by PPA. Specifically, PPA can protect adolescents who suffer from low-to-moderate cybervictimization caused by depression, which is in line with the reverse risk-buffering model (Kobasa and Puccetti, 1983). This result is partially consistent with our hypothesis 2 and the predictions derived from the risk and resilience framework (Masten, 2001). A possible explanation is that prosocial peers can provide immediate emotional social support and constructive suggestions for solving problems when adolescents experience cybervictimization, which in turn reduces the risk of depression (Birkeland et al., 2014). However, the protective effect of PPA did not operate under high levels of cybervictimization. That is, due to the highly traumatic nature of cybervictimization, it may be difficult for adolescents who experience cybervictimization to have positive outcomes even if they have high levels of PPA. Two possible explanations may be considered for this finding. First, high levels of cybervictimization can make victims distrust others, which may contribute to their social isolation (Calvete et al., 2016). In this case, adolescents are less likely to seek help and obtain support from their prosocial peers. Second, some researchers suggest that cybervictimization has a “snowball effect” (Tokunaga, 2010). With repeated occurrences of cybervictimization, adolescent may shift from external attributions to self-blaming attributions, which lead to higher level of hopelessness and in turn, increases their risk for depression (Slonje et al., 2013; Chu et al., 2018). In view of the above two reasons, high levels of cybervictimization are especially harmful to adolescents, which may make the protection of PPA insufficient to offset the risk of cybervictimization. As a result, we should recognize that the protective effect of PPA is limited and targeted preventive interventions should be conducted for adolescents who suffer from cybervictimization.

This study also found that PPA does not moderate the relationship between depression and adolescents’ IA. This result suggests that PPA does not cushion the adverse impact of depression on adolescents’ IA. One possible explanation is that depression is a very strong predictor of IA (Vondrackova and Gabrhelik, 2016). In particular, depression can not only directly predict IA but the complications of depression, such as attention problems, social avoidance, and low self-esteem, are also highly correlated with IA (Lam, 2014; Stavropoulos et al., 2019). In addition, a meta-analysis showed that intrapersonal variables have a statistically greater impact on IA than interpersonal variables (Koo and Kwon, 2014). Therefore, the protective effect of PPA may not be enough to offset the risk of IA from depression.

Moreover, as a supportive but non-directive relationship, PPA may not provide critical buffering resources (such as behavior monitoring and emotion regulation skills) to prevent IA for adolescents with high levels of depression (Vondrackova and Gabrhelik, 2016). Considering the above reasons, it is necessary for future research to explore the moderating role of other important protective factors (such as parent-child communication and teacher-student relationship) in the relationship between depression and IA.

### Limitations and Future Directions

The current study has several limitations that should be acknowledged. First, our study used a cross-sectional study design. This does not allow us to determine the causal direction between variables. Future studies should establish longitudinal model or use experimental designs to identify the causal relationships. Second, this study used adolescent self-reporting measures to collect data, which might have involved self-presentation and recall biases (Williams et al., 1989), which might have adversely affected the validity of the study. In future studies, multiple informants (e.g., peer, parent, and teacher reports) will be valuable in strengthening the reliability of the findings. Third, this study found that depression partially mediates the relationship between cybervictimization and IA, and PPA only moderates the link between cybervictimization and depression. Thus, other mediators and moderators should be considered in future studies to supplement the research results. Finally, the current study used a convenient sample from junior middle schools in south China, which does not be represent the larger Chinese population and adolescents residing in other regions of China. As a result, the catholicity of the results should be further verified by a cross-regional sample.

### Implications for Practice

Despite these limitations, the results of our study have several important implications for the practice of prevention and intervention of adolescents’ IA. First, considering that cybervictimization is positively correlated with depression and IA, reducing the incidence of cybervictimization may be an effective way to improve the mental health of adolescents. Second, this study indicated that depression may be an important mediating mechanism between cybervictimization and adolescents’ IA. This suggests that identifying and paying attention to adolescents with high depression may help educate practitioners to improve the efficiency of adolescents’ IA interventions. Third, our research showed that PPA may help protect adolescents against the development of depression associated with cybervictimization. Therefore, encouraging adolescents to participate in prosocial activities and affiliating with prosocial peers could help prevent depression, and thus prevent IA.

## Data Availability Statement

The raw data supporting the conclusions of this article will be made available by the corresponding author, without undue reservation.

## Ethics Statement

The studies involving human participants were reviewed and approved by Ethics Review Committee of the School of Education, Guangzhou University. Written informed consent to participate in this study was provided by the participants’ legal guardian/next of kin.

## Author Contributions

ZW, CW, CY, JW, and WZ conceived and designed the research. CW, CY, and JW performed the research. CW, CY, and SZ analyzed the data. ZW, QX, MX, CW, CY, SZ, SL, JW, and WZ contributed to the writing of the manuscript. ZW, QX, MX, CW, CY, SZ, SL, JW, and WZ revised the paper critically for important intellectual content, commented on, and approved the final manuscript. All authors contributed to the article and approved the submitted version.

### Conflict of Interest

The authors declare that the research was conducted in the absence of any commercial or financial relationships that could be construed as a potential conflict of interest.

## References

[ref1] Alimoradi Z. Lin C. Y. Brostrom A. Bulow P. H. Bajalan Z. Griffiths M. D.. (2019). Internet addiction and sleep problems: a systematic review and meta-analysis. Sleep Med. Rev. 47, 51–61. 10.1016/j.smrv.2019.06.004, PMID: 31336284

[ref2] Anand N. Thomas C. Jain P. A. Bhat A. Thomas C. Prathyusha P. V.. (2018). Internet use behaviors, internet addiction and psychological distress among medical college students: a multi centre study from South India. Asian J. Psychiatr. 37, 71–77. 10.1016/j.ajp.2018.07.020, PMID: 30145540

[ref3] Aricak O. T. Ozbay A. (2016). Investigation of the relationship between cyberbullying, cybervictimization, alexithymia and anger expression styles among adolescents. Comput. Hum. Behav. 55, 278–285. 10.1016/j.chb.2015.09.015

[ref4] Baldry A. C. Sorrentino A. Farrington D. P. (2019). Cyberbullying and cybervictimization versus parental supervision, monitoring and control of adolescents' online activities. Child Youth Serv. Rev. 96, 302–307. 10.1016/j.childyouth.2018.11.058

[ref5] Bandura A. (1977). Social learning theory. Oxford: Prentice-Hall.

[ref6] Bao J. M. Li H. H. Song W. Jiang S. Y. (2020). Being bullied, psychological pain and suicidal ideation among Chinese adolescents: a moderated mediation model. Child Youth Serv. Rev. 109:104744. 10.1016/j.childyouth.2020.104744

[ref7] Barry C. M. Wentzel K. R. (2006). Friend influence on prosocial behavior: the role of motivational factors and friendship characteristics. Dev. Psychol. 42, 153–163. 10.1037/0012-1649.42.1.153, PMID: 16420125

[ref8] Birkeland M. S. Breivik K. Wold B. (2014). Peer acceptance protects global self-esteem from negative effects of low closeness to parents during adolescence and early adulthood. J. Youth Adolesc. 43, 70–80. 10.1007/s10964-013-9929-1, PMID: 23435859 PMC3889815

[ref9] Block J. J. (2008). Issues for DSM-V: internet addiction. Am. J. Psychiatr. 165, 306–307. 10.1176/appi.ajp.2007.07101556, PMID: 18316427

[ref45] Brochado S. Soares S. Fraga S. (2017). A scoping review on studies of cyberbullying prevalence among adolescents. Trauma Violence and Abuse 18, 523–531. 10.1177/1524838016641668 27053102

[ref10] Burt S. A. Klump K. L. (2014). Prosocial peer affiliation suppresses genetic influences on non-aggressive antisocial behaviors during childhood. Psychol. Med. 44, 821–830. 10.1017/S0033291713000974, PMID: 23659437 PMC3749251

[ref11] Calvete E. Orue I. Gamez-Guadix M. (2016). Cyberbullying victimization and depression in adolescents: the mediating role of body image and cognitive schemas in a one-year prospective study. Eur. J. Crim. Policy Res. 22, 271–284. 10.1007/s10610-015-9292-8

[ref12] Cenat J. M. Blais M. Lavoie F. Caron P. -O. Hebert M. (2018). Cyberbullying victimization and substance use among Quebec high schools students: the mediating role of psychological distress. Comput. Hum. Behav. 89, 207–212. 10.1016/j.chb.2018.08.014

[ref13] Chang F. C. Chiu C. H. Miao N. F. Chen P. H. Lee C. M. Chiang J. T.. (2015). The relationship between parental mediation and internet addiction among adolescents, and the association with cyberbullying and depression. Compr. Psychiatry 57, 21–28. 10.1016/j.comppsych.2014.11.013, PMID: 25487108

[ref14] Cheng Y. S. Tseng P. T. Lin P. Y. Chen T. Y. Stubbs B. Carvalho A. F.. (2018). Internet addiction and its relationship with suicidal behaviors: a meta-analysis of multinational observational studies. J. Clin. Psychiatry 79:17r11761. 10.4088/JCP.17r11761, PMID: 29877640

[ref15] China Internet Network Information Center [CNNIC] (2019). The 44th statistical report on internet development in China. Available at: http://www.cnnic.cn/ (Accessed March 12, 2020).

[ref16] Chu X. -W. Fan C. -Y. Liu Q. -Q. Zhou Z. -K. (2018). Cyberbullying victimization and symptoms of depression and anxiety among Chinese adolescents: examining hopelessness as a mediator and self-compassion as a moderator. Comput. Hum. Behav. 86, 377–386. 10.1016/j.chb.2018.04.039

[ref17] Cole D. A. Zelkowitz R. L. Nick E. Martin N. C. Roeder K. M. Sinclair-McBride K.. (2016). Longitudinal and incremental relation of cybervictimization to negative self-cognitions and depressive symptoms in young adolescents. J. Abnorm. Child Psychol. 44, 1321–1332. 10.1007/s10802-015-0123-7, PMID: 26747449 PMC4938781

[ref18] Cyders M. A. Littlefield A. K. Coffey S. Karyadi K. A. (2014). Examination of a short English version of the UPPS-P impulsive behavior scale. Addict. Behav. 39, 1372–1376. 10.1016/j.addbeh.2014.02.013, PMID: 24636739 PMC4055534

[ref19] Erdur-Baker O. Kavsut F. (2007). Cyber bullying: a new face of peer bullying. Eurasian J. Educ. Res. 7, 31–42.

[ref20] Fabes R. A. Hanish L. D. Martin C. L. Moss A. Reesing A. (2012). The effects of young children’s affiliations with prosocial peers on subsequent emotionality in peer interactions. Br. J. Dev. Psychol. 30, 569–585. 10.1111/j.2044-835X.2011.02073.x, PMID: 23039333 PMC3466482

[ref21] Fisher B. W. Gardella J. H. Teurbe-Tolon A. R. (2016). Peer cybervictimization among adolescents and the associated internalizing and externalizing problems: a meta-analysis. J. Youth Adolesc. 45, 1727–1743. 10.1007/s10964-016-0541-z, PMID: 27447707

[ref22] Gamez-Guadix M. Calvete E. Orue I. Las Hayas C. (2015). Problematic internet use and problematic alcohol use from the cognitive-behavioral model: a longitudinal study among adolescents. Addict. Behav. 40, 109–114. 10.1016/j.addbeh.2014.09.009, PMID: 25244690

[ref23] Gamez-Guadix M. Orue I. Smith P. K. Calvete E. (2013). Longitudinal and reciprocal relations of cyberbullying with depression, substance use, and problematic internet use among adolescents. J. Adolesc. Health 53, 446–452. 10.1016/j.jadohealth.2013.03.030, PMID: 23721758

[ref24] Gao T. T. Meng X. F. Qin Z. Y. Zhang H. Gao J. L. Kong Y. X.. (2018). Association between parental marital conflict and internet addiction: a moderated mediation analysis. J. Affect. Disord. 240, 27–32. 10.1016/j.jad.2018.07.005, PMID: 30048833

[ref25] Guo S. (2016). A meta-analysis of the predictors of cyberbullying perpetration and victimization. Psychol. Sch. 53, 432–453. 10.1002/pits.21914

[ref26] Han S. C. Margolin G. (2016). Intergenerational links in victimization: prosocial friends as a buffer. J. Child Adolesc. Trauma 9, 153–165. 10.1007/s40653-015-0075-7, PMID: 27429687 PMC4943841

[ref27] Healy K. L. Sanders M. R. (2018). Mechanisms through which supportive relationships with parents and peers mitigate victimization, depression and internalizing problems in children bullied by peers. Child Psychiatry Hum. Dev. 49, 800–813. 10.1007/s10578-018-0793-9, PMID: 29473091

[ref28] Khantzian E. J. (1985). The self-medication hypothesis of addictive disorders – focus on heroin and cocaine dependence. Am. J. Psychiatry 142, 1259–1264. 10.1176/ajp.142.11.1259, PMID: 3904487

[ref53] Kobasa S. C. Puccetti M. C. (1983). Personality and social resources in stress resistance. J. Pers. Soc. Psychol. 45, 839–850. 10.1037/0022-3514.45.4.839 6631665

[ref29] Koo H. J. Kwon J. H. (2014). Risk and protective factors of internet addiction: a meta-analysis of empirical studies in Korea. Yonsei Med. J. 55, 1691–1711. 10.3349/ymj.2014.55.6.1691 25323910 PMC4205713

[ref30] Kowalski R. M. Giumetti G. W. Schroeder A. N. Lattanner M. R. (2014). Bullying in the digital age: a critical review and meta-analysis of cyberbullying research among youth. Psychol. Bull. 140, 1073–1137. 10.1037/a0035618, PMID: 24512111

[ref31] Kowalski R. Limber S. McCord A. (2018). A developmental approach to cyberbullying: prevalence and protective factors. Aggress. Violent Behav. 45, 20–32. 10.1016/j.avb.2018.02.009

[ref32] Kwan I. Dickson K. Richardson M. MacDowall W. Burchett H. Stansfield C.. (2020). Cyberbullying and children and young people’s mental health: a systematic map of systematic reviews. Cyberpsychol. Behav. Soc. Netw. 23, 72–82. 10.1089/cyber.2019.0370, PMID: 31977251 PMC7044782

[ref33] Lam L. T. (2014). Internet gaming addiction, problematic use of the internet, and sleep problems: a systematic review. Curr. Psychiatry Rep. 16:444. 10.1007/s11920-014-0444-1, PMID: 24619594

[ref34] Landoll R. R. La Greca A. M. Lai B. S. Chan S. F. Herge W. M. (2015). Cyber victimization by peers: prospective associations with adolescent social anxiety and depressive symptoms. J. Adolesc. 42, 77–86. 10.1016/j.adolescence.2015.04.002, PMID: 25938204

[ref35] Lee J. Hwang J. Y. Park S. M. Jung H. Y. Choi S. W. Kim D. J.. (2014). Differential resting-state EEG patterns associated with comorbid depression in internet addiction. Prog. Neuro-Psychopharmacol. Biol. Psychiatry 50, 21–26. 10.1016/j.pnpbp.2013.11.016, PMID: 24326197

[ref36] Li Y. Li D. P. Li X. Zhou Y. Y. Sun W. Q. Wang Y. H. (2018). Cyber victimization and adolescent depression: the mediating role of psychological insecurity and the moderating role of perceived social support. Child Youth Serv. Rev. 94, 10–19. 10.1016/j.childyouth.2018.09.027

[ref37] Lin L. Liu J. B. Cao X. L. Wen S. Y. Xu J. C. Xue Z. P.. (2020). Internet addiction mediates the association between cyber victimization and psychological and physical symptoms: moderation by physical exercise. BMC Psychiatry 20:144. 10.1186/s12888-020-02548-6, PMID: 32245443 PMC7118978

[ref38] Mason M. Mennis J. Russell M. Moore M. Brown A. (2019). Adolescent depression and substance use: the protective role of prosocial peer behavior. J. Abnorm. Child Psychol. 47, 1065–1074. 10.1007/s10802-018-0501-z, PMID: 30547314 PMC6788757

[ref39] Masten A. S. (2001). Ordinary magic: resilience processes in development. Am. Psychol. 56, 227–238. 10.1037/0003-066x.56.3.227, PMID: 11315249

[ref40] Metzler C. W. Biglan A. Ary D. V. Fuzhong L. (1998). The stability and validity of early adolescents’ reports of parenting constructs. J. Fam. Psychol. 12, 600–619. 10.1037/0893-3200.12.4.600

[ref41] Palermiti A. L. Servidio R. Bartolo M. G. Costabile A. (2017). Cyberbullying and self-esteem: an Italian study. Comput. Hum. Behav. 69, 136–141. 10.1016/j.chb.2016.12.026

[ref42] Pontes H. M. Griffiths M. D. (2015). Measuring DSM-5 internet gaming disorder: development and validation of a short psychometric scale. Comput. Hum. Behav. 45, 137–143. 10.1016/j.chb.2014.12.006

[ref43] Radloff L. S. (1977). The CES-D scale: a self-report depression scale for research in the general population. Appl. Psychol. Meas. 1, 385–401. 10.1177/014662167700100306

[ref44] Rusby J. C. Mason M. Gau J. M. Westling E. Light J. M. Mennis J.. (2019). Relational victimization and peer affiliate prosocial behaviors in African American adolescents: moderating effects of gender and antisocial behavior. J. Adolesc. 71, 91–98. 10.1016/j.adolescence.2019.01.002, PMID: 30654276 PMC6946022

[ref46] Sela Y. Zach M. Amichay-Hamburger Y. Mishali M. Omer H. (2020). Family environment and problematic internet use among adolescents: the mediating roles of depression and fear of missing out. Comput. Hum. Behav. 106:106226. 10.1016/j.chb.2019.106226

[ref47] Slonje R. Smith P. K. Frisen A. (2013). The nature of cyberbullying, and strategies for prevention. Comput. Hum. Behav. 29, 26–32. 10.1016/j.chb.2012.05.024

[ref48] Sourander A. Klomek A. B. Ikonen M. Lindroos J. Luntamo T. Koskelainen M.. (2010). Psychosocial risk factors associated with cyberbullying among adolescents: a population-based study. Arch. Gen. Psychiatry 67, 720–728. 10.1001/archgenpsychiatry.2010.79, PMID: 20603453

[ref49] Spada M. M. (2014). An overview of problematic internet use. Addict. Behav. 39, 3–6. 10.1016/j.addbeh.2013.09.007, PMID: 24126206

[ref50] Stavropoulos V. Adams B. L. M. Beard C. L. Dumble E. Trawley S. Gomez R.. (2019). Associations between attention deficit hyperactivity and internet gaming disorder symptoms: is there consistency across types of symptoms, gender and countries? Addict. Behav. Rep. 9:100158. 10.1016/j.abrep.2018.100158, PMID: 30671530 PMC6327637

[ref65] Teddlie C. Yu F. (2007). Mixed methods sampling a typology with examples. J. Mix. Methods Res. 1, 77–100. 10.1177/2345678906292430

[ref51] Tian Y. L. Yu C. F. Lin S. Lu J. M. Liu Y. Zhang W. (2019). Parental psychological control and adolescent aggressive behavior: deviant peer affiliation as a mediator and school connectedness as a moderator. Front. Psychol. 10:358. 10.3389/fpsyg.2019.00358, PMID: 30846957 PMC6393334

[ref52] Tokunaga R. S. (2010). Following you home from school: a critical review and synthesis of research on cyberbullying victimization. Comput. Hum. Behav. 26, 277–287. 10.1016/j.chb.2009.11.014

[ref54] Vondrackova P. Gabrhelik R. (2016). Prevention of internet addiction: a systematic review. J. Behav. Addict. 5, 568–579. 10.1556/2006.5.2016.085, PMID: 27998173 PMC5370363

[ref55] Walden B. Mcgue M. Lacono W. G. Burt S. A. Elkins I. (2004). Identifying shared environmental contributions to early substance use: the respective roles of peers and parents. J. Abnorm. Psychol. 113, 440–450. 10.1037/0021-843x.113.3.440, PMID: 15311989

[ref56] Wang W. Xie X. Wang X. Lei L. Hu Q. Jiang S. (2019). Cyberbullying and depression among Chinese college students: a moderated mediation model of social anxiety and neuroticism. J. Affect. Disord. 256, 54–61. 10.1016/j.jad.2019.05.061, PMID: 31158716

[ref57] Williams L. J. Cote J. A. Buckley M. R. (1989). Lack of method variance in self-reported affect and perceptions at work: reality or artifact? J. Appl. Psychol. 74, 462–468.

[ref58] Wright M. (2018). Cyberbullying victimization through social networking sites and adjustment difficulties: the role of parental mediation. J. Assoc. Inf. Syst. 19, 113–123. 10.17705/jais1.00486

[ref59] Yen J. -Y. Lin H. -C. Chou W. -P. Liu T. -L. Ko C. -H. (2019). Associations among resilience, stress, depression, and internet gaming disorder in young adults. Int. J. Environ. Res. Public Health 16:3181. 10.3390/ijerph16173181, PMID: 31480445 PMC6747224

[ref60] Young K. S. De Abreu C. N. (2011). Internet addiction: A handbook and guide to evaluation and treatment. Hoboken, NJ: John Wiley.

[ref61] Yucens B. Uzer A. (2018). The relationship between internet addiction, social anxiety, impulsivity, self-esteem, and depression in a sample of Turkish undergraduate medical students. Psychiatry Res. 267, 313–318. 10.1016/j.psychres.2018.06.033 29957547

[ref62] Zhao F. Zhang Z. H. Bi L. D. Wu X. S. Wang W. J. Li Y. F. (2017). The association between life events and internet addiction among Chinese vocational school students: the mediating role of depression. Comput. Hum. Behav. 70, 30–38. 10.1016/j.chb.2016.12.057

[ref63] Zhu J. Yu C. Zhang W. Bao Z. Jiang Y. Chen Y.. (2016). Peer victimization, deviant peer affiliation and impulsivity: predicting adolescent problem behaviors. Child Abuse Negl. 58, 39–50. 10.1016/j.chiabu.2016.06.008, PMID: 27348798

[ref64] Zych I. Farrington D. P. Ttofi M. M. (2019). Protective factors against bullying and cyberbullying: a systematic review of meta-analyses. Aggress. Violent Behav. 45, 4–19. 10.1016/j.avb.2018.06.008

